# Expression Profiles of Five Common Cancer Membrane Protein Antigens Collected for the Development of Cocktail CAR-T Cell Therapies Applicable to Most Solid Cancer Patients

**DOI:** 10.3390/ijms26052145

**Published:** 2025-02-27

**Authors:** Tetsuya Nakatsura, Kazumasa Takenouchi, Jun Kataoka, Yusuke Ito, Sae Kikuchi, Hiroki Kinoshita, Kazunobu Ohnuki, Toshihiro Suzuki, Nobuo Tsukamoto

**Affiliations:** Division of Cancer Immunotherapy, Exploratory Oncology Research and Clinical Trial Center, National Cancer Center, Kashiwa 277-8577, Japan; katakeno@east.ncc.go.jp (K.T.); jkataoka@east.ncc.go.jp (J.K.); yusito@east.ncc.go.jp (Y.I.); sakikuc4@east.ncc.go.jp (S.K.); hirokino@east.ncc.go.jp (H.K.); konuki@east.ncc.go.jp (K.O.); toshsuzu@east.ncc.go.jp (T.S.); nobutsuk@east.ncc.go.jp (N.T.)

**Keywords:** common cancer antigens, CAR-T cell therapy, membrane protein, solid cancer, glypican-3, ROBO1, EphB4, CLDN1, LAT1, cocktail CAR-T

## Abstract

Although CD19 CAR-T has been highly effective against B-cell blood cancers, there are few reports of successful treatments for solid cancers, probably because there are few protein antigens specifically expressed on the surface of the cancer cell membrane. The key to developing a groundbreaking CAR-T cell therapy effective against solid cancers is to “overcome the heterogeneity of cancer antigens”. For this purpose, it is necessary to target multiple cancer antigens simultaneously. In this study, we performed immunohistochemical analysis of various solid cancer specimens using antibodies against ROBO1, EphB4, CLDN1, and LAT1 in addition to GPC3, which we have previously studied. These antigens were frequently expressed in various solid cancers but shown to be rarely expressed, with some exceptions, in non-cancerous normal organs adjacent to the cancer. Although ROBO1 and GPC3 are often expressed in cytoplasm, there are also cases in which they are expressed on the cell membrane depending on the type of cancer. On the other hand, it has been revealed that three antigens—EphB4, CLDN1, and LAT1—are frequently expressed only on the cell membrane of cancer cells in various solid cancers, suggesting that they may be ideal targets for CAR-T cell therapy.

## 1. Introduction

We discovered the cancer-specific antigen glypican-3 (GPC3) and identified peptides that can induce cytotoxic T lymphocytes (CTLs) in an HLA-A24- or -A2-restricted manner and conducted clinical trials of peptide vaccines for hepatocellular carcinoma, ovarian clear cell carcinoma, and pediatric cancer [1,2,3,4,5]. We have experienced successful cases even in advanced cancers, demonstrating the possibility of preventing recurrence. Seven children with childhood cancer who received this vaccine have still been growing healthily and without recurrence for about 10 years. We are currently identifying T cell receptors (TCRs) from the peptide-specific CTL clones of this girl who overcame hepatoblastoma and developing TCR-T cell therapy. GPC3 is expressed in normal organs such as the placenta, fetal liver, kidneys, and lungs but rarely in normal organs in adults. It is an extremely safe cancer antigen, but unfortunately, it is only expressed in a limited number of cancer types.

While CTL and TCR-T cell therapy target peptides derived from cancer antigens presented by HLA class I on the surface of cancer cells, chimeric antigen receptor (CAR)-T cell therapy targets membrane protein antigens on cancer cells and is not HLA-restricted. CAR-T cell therapy targeting CD19 and BCMA has remarkably treated blood cancers [6,7,8]. On the other hand, a wide variety of CAR-T cell therapies targeting Her2 [9,10], GD2 [10,11], mesothelin (MSLN) [10,12], CLDN18.2 [10,13], etc., for solid cancers are being developed. However, there are still a few promising results that have been reported.

GPC3 is also a membrane protein, and CAR-T cell therapy targeting GPC3 has been developed worldwide [10,14], but dramatic efficacy has not yet been reported. Our immunohistochemical analysis has shown that GPC3 is expressed in many hepatocellular carcinomas, but the expression pattern on the cell membrane is rare. To increase the effectiveness of CAR-T cell therapy, the antigen must be properly expressed on the cell membrane of most cancer cells. Cancer has heterogeneity, and it is not possible to eradicate cancer by targeting only one antigen. Therefore, in this study, we challenged ourselves to collect some common cancer membrane protein antigens, including GPC3, expressed in a wide range of solid cancers but only in limited amounts in normal tissues. To be a promising target for CAR-T cell therapy, the antigen must be expressed on the cell membrane of cancer cells. In this study, we added roundabout guidance receptor 1 (ROBO1) [15,16,17], Eph receptor B4 (EphB4) [18,19,20,21,22,23], claudin-1 (CLDN1) [24,25,26,27], and L-type amino acid transporter 1 (LAT1) [28,29,30,31] to GPC3, which we have studied so far. We verified the expression of these five common cancer antigens on the cell membrane of cancer cells by immunohistochemical analysis of tissue sections, including cancerous and non-cancerous areas of various solid cancers, to find combinations of these common cancer membrane protein antigens that are promising targets for CAR-T cell therapy targeting all solid cancers.

## 2. Results

### 2.1. Representative Examples of Plasma Membrane Expression of Five Common Cancer Antigens in Cancer Cells

**Figure 1:**
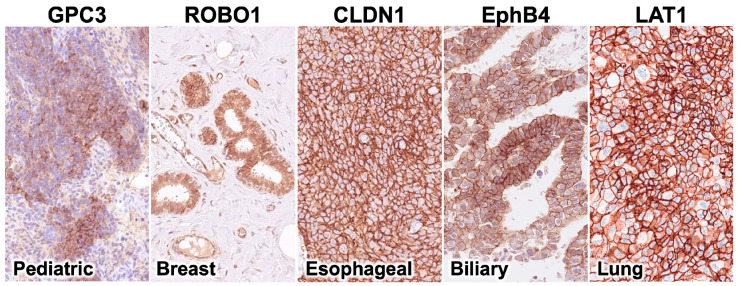
Representative examples of plasma membrane expression of five common cancer antigens in cancer cells. Representative examples of five common cancer antigens, GPC3, ROBO1, CLDN1, EphB4, and LAT1, expressed on the cell membrane of cancer cells are shown.

Representative examples of five common cancer antigens, GPC3, ROBO1, CLDN1, EphB4, and LAT1, expressed on the cell membrane of cancer cells are shown (Figure 1). CLDN1, EphB4, and LAT1 were expressed only on the cell membrane of cancer cells. In contrast, GPC3 and ROBO1 were mainly expressed in the cytoplasm of cancer cells, although some expression patterns on the cell membrane of cancer cells were observed.

### 2.2. Frequency of Expression of Five Common Cancer-Specific Antigens as Cell Membrane Proteins in Various Solid Cancers

**Figure 2:**
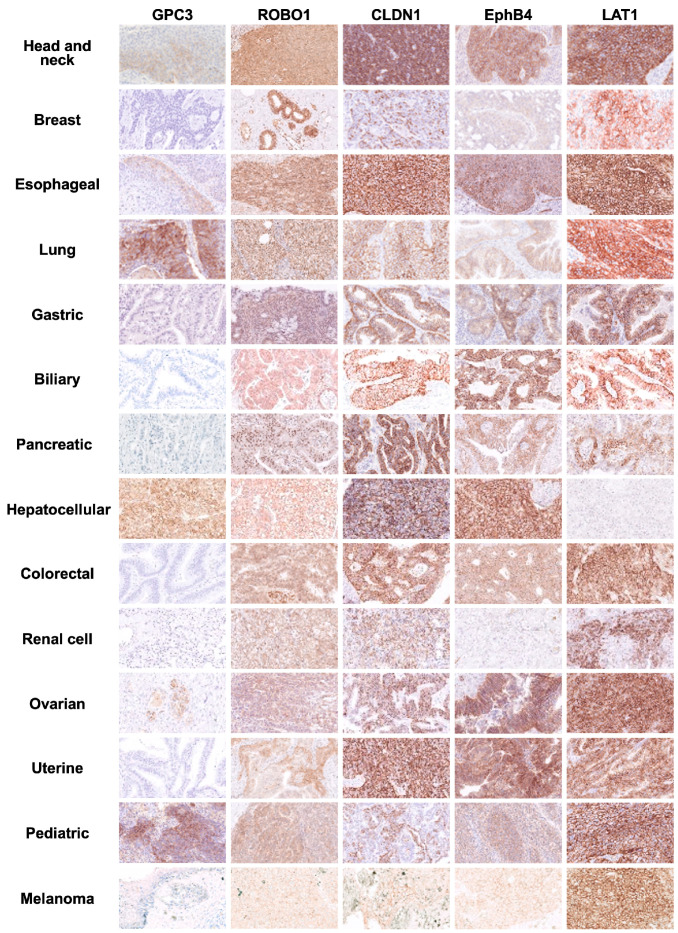
Expression of 5 common cancer-specific antigens in various solid cancers and their expression as cell membrane proteins. Representative examples of the expression patterns of 5 common cancer-specific antigens in each cancer type by their intracellular localization (cell membrane, cytoplasm) in cancer tissues are shown.

Immunostaining was performed using formalin-fixed paraffin sections, including cancerous and non-cancerous areas, from a total of 385 cases of advanced cancer that had been surgically resected. The breakdown of the 385 cases is as follows: 57 cases of head and neck cancer (21 oral cancer, 36 pharyngeal cancer), 36 breast cancer, 16 esophageal squamous cell carcinoma, 72 lung cancer (45 adenocarcinoma, 27 squamous cell carcinoma), 29 gastric cancer (including 19 endoscopically resected early stage 1 gastric cancer), 20 hepatocellular carcinoma, 30 biliary tract cancer (10 intrahepatic bile duct cancer, 10 extrahepatic bile duct cancer, 10 gallbladder cancer), 13 pancreatic cancer, 15 colorectal cancer, 19 renal cell carcinoma, 18 ovarian cancer (17 serous adenocarcinoma, 1 clear cell carcinoma), 7 uterine cancer, 20 melanoma (7 ALM, 5 mucosal mucous, 5 SSM, 3 LMM), 33 childhood cancer (9 hepatoblastoma, 8 neuroblastoma, 10 nephroblastoma: Wilms tumor, 6 germ cell tumor) (Figure 2, Table 1).

A feature of this study is that we conducted a large-scale investigation of the expression of these five membrane protein antigens on the cell membrane (Table 1). The positive rate of GPC3 expression on the cell membrane was only 5% in hepatocellular carcinoma, 33% in hepatoblastoma, 50% in nephroblastoma: Wilms tumor, 17% in germ cell tumor, 11% in lung squamous cell carcinoma, one case of ovarian clear cell carcinoma, 8% in pharyngeal cancer, and 6% in esophageal cancer, as well as one case each of ovarian serous adenocarcinoma and lung adenocarcinoma. Overall, this sharply declined to 5%. ROBO1 was also expressed on the cell membrane in 37% of pharyngeal cancer, 14% of oral cancer, 6% of breast cancer, 38% of esophageal cancer, 52% of lung squamous cell carcinoma, 11% of lung adenocarcinoma, 21% of biliary tract cancer, 74% of hepatocellular carcinoma, 11% of renal cell carcinoma, 14% of uterine cancer, 67% of hepatoblastoma, 80% of nephroblastoma, 17% of germ cell tumor, 14% of neuroblastoma, and 10% of melanoma. The overall positive rate of expression on the cell membrane dropped to 22%. However, it was revealed that ROBO1 is frequently expressed on the cell membrane in hepatocellular carcinoma, pediatric cancer, lung squamous cell carcinoma, pharyngeal cancer, and esophageal cancer, suggesting that CAR-T cell therapy targeting ROBO1 may be effective against these cancer types.

The greatest achievement of this study was demonstrating that three kinds of cell membrane-expressing proteins—CLDN1, EphB4, and LAT1—are frequently expressed in various types of solid cancers (Figure 2, Table 1). CLDN1 was positive in 100% of oral and pharyngeal cancer, 94% of esophageal cancer, 93% of lung squamous cell carcinoma, 80% of gastric cancer, 93% of biliary tract cancer, 100% of pancreatic cancer, 100% of colorectal cancer, 94% of ovarian cancer, 100% of uterine cancer, and 100% of hepatoblastoma, as well as 65% of hepatocellular carcinoma, 44% of lung adenocarcinoma, 40% of nephroblastoma: Wilms tumor, and 33% of germ cell tumor. An interesting finding was that the positive rate for advanced gastric cancer was 80%, whereas the positive rate for early-stage cancer was only 32%. On the other hand, the positive rate was 25% for melanoma, 16% for renal cell carcinoma, 20% for breast cancer, and 0% for neuroblastoma, resulting in an overall positive rate of 64%. Next, EphB4 showed high positive rates in 100% of oral cancer, 92% of pharyngeal cancer, 83% of breast cancer, 94% of esophageal cancer, 79% of lung cancer, 100% of gastric cancer (including 89% of early gastric cancer), 80% of biliary tract cancer, 75% of pancreatic cancer, 70% of hepatocellular carcinoma, 93% of colorectal cancer, 83% of ovarian cancer, 86% of uterine cancer, 89% of hepatoblastoma, and 65% of melanoma. The positive rates were 30% in nephroblastoma, 33% in germ cell tumor, and 0% in renal cell carcinoma and neuroblastoma, resulting in an overall positive rate of 76%. Finally, the positive rates of LAT1 were 100% for oral cancer, 90% for pharyngeal cancer, 94% for esophageal cancer, 100% for lung squamous cell carcinoma, 80% for gastric cancer, 100% for colorectal cancer, 67% for ovarian cancer, 100% for uterine cancer, 100% for germ cell tumor, 55% for melanoma, 50% for biliary tract cancer, 49% for lung adenocarcinoma, 42% for breast cancer, 38% for neuroblastoma, 31% for pancreatic cancer, and 30% for nephroblastoma. An interesting finding is that the positivity rate of LAT1 is low, at 21% in early-stage gastric cancer compared to 80% in advanced gastric cancer. The other positive rates were 22% for hepatoblastoma, 16% for renal cell carcinoma, and 0% for hepatocellular carcinoma, resulting in an overall positive rate of 58%.

**Figure 3:**
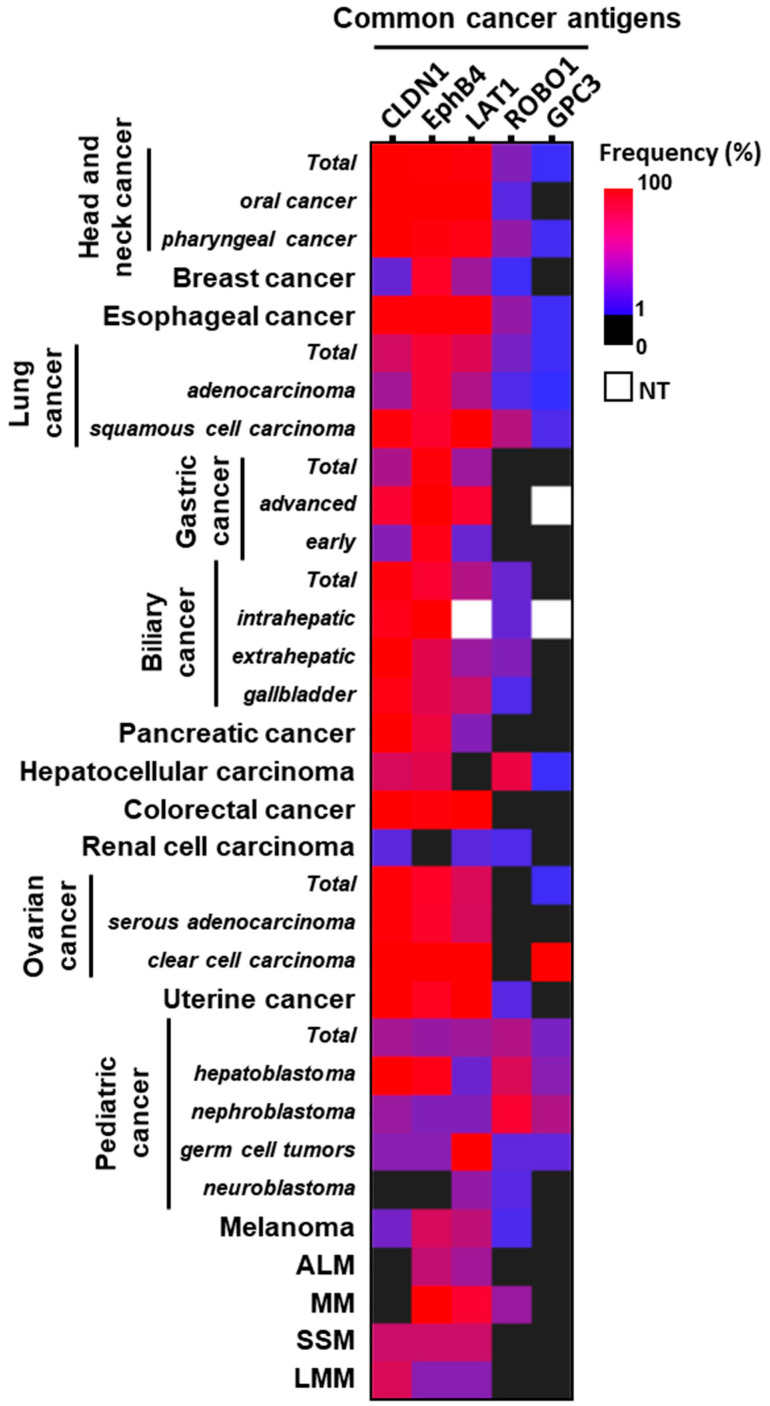
Heatmap of expression frequency of five common cancer antigens on the cell membrane in various solid cancers. The expression frequency values in Table 1 are shown in a heatmap, with 1% in blue and 100% in red. The closer to red, the higher the expression frequency, and the closer to blue, the lower the expression frequency. Black is 0%, meaning no expression with no positive cases. White is not tested: NT.

Since the list of numbers is difficult to understand, a heat map was created to make the results in Table 1 more straightforward visually (Figure 3); 1% is blue, and 100% is red. The closer to red, the higher the expression frequency, and the closer to blue, the lower the expression frequency. Black is 0%, which means there are no positive cases and no expression. White is not tested (NT). Renal cell carcinoma is the only cancer in which the expression frequency of all five types is low, but all other cancers have high expression frequencies of multiple antigens. In particular, EphB4, CLDN1, and LAT1 are expressed at high frequencies in most cancers, with a few exceptions. In neuroblastoma, EphB4 and CLDN1 are not expressed, but in addition to LAT1, some ROBO1 is expressed. In breast cancer, the expression frequency of CLDN1 is low, but in addition to EphB4, LAT1 is expressed at high frequencies. In hepatocellular carcinoma, LAT1 is not expressed, but instead, ROBO1 is expressed at high frequencies. ROBO1 and GPC3 are also expressed on the cell membrane in some types of cancer, and the five antigens also cover various pediatric cancers.

### 2.3. Expression of Five Common Cancer Membrane Protein Antigens in Non-Cancerous Normal Organs Adjacent to Various Cancers

**Figure 4:**
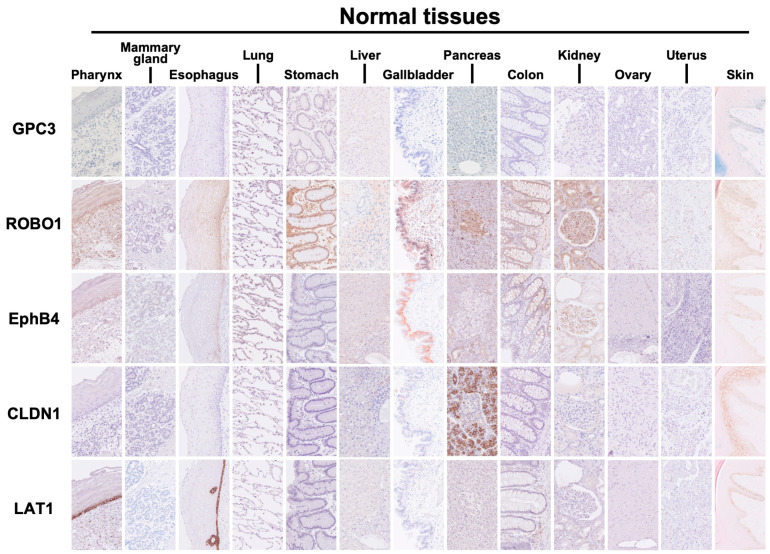
Expression of 5 common cancer-specific antigens in non-cancerous normal organs adjacent to various cancers. Representative examples of the expression patterns of 5 common cancer-specific antigens in non-cancerous normal organs (pharynx, mammary gland, esophagus, lung, stomach, liver, gallbladder, pancreas, colon, kidney, ovary, uterus, and skin) adjacent to the cancer lesions of a total of 385 cases of surgically resected advanced cancer are shown.

Figure 4 shows representative examples of the expression patterns of five common cancer membrane protein antigens in non-cancerous normal organs (pharynx, mammary gland, esophagus, lung, stomach, liver, gallbladder, pancreas, colon, kidney, ovary, uterus, and skin) adjacent to the cancer lesions of a total of 385 cases of surgically resected advanced cancer as described above. Our previous study showed that GPC3 is expressed in the placenta and fetal liver, kidney, and lung but is rarely expressed in normal adult organs, except for weak expression in renal tubular cells. Similar results were obtained this time as well. ROBO1 staining was observed in the basal layer cells of the squamous epithelium of the pharynx and esophagus and in the mucosal epithelium cells of the stomach and gallbladder, with staining that was clearly weaker than that in the cancerous areas. EphB4 staining was observed in the basal layer cells of the squamous epithelium of the pharynx and esophagus and in the mucosal epithelial cells of the stomach, gallbladder, and large intestine. However, the staining was clearly weaker than in the cancerous areas. CLDN1 staining was observed in the acinar cells of the pancreas and skin, although it was weaker than in the cancerous area, and moderate staining was observed. LAT1 staining was observed in the cells of the basal layer of the squamous epithelium of the pharynx and esophagus, which was relatively strong. When performing treatments such as CAR-T cell therapy involving antibodies targeting ROBO1, EphB4, CLDN1, and LAT1, it is necessary to be careful of adverse events in normal organs where these antigens are expressed.

### 2.4. Development of a Companion Diagnostic Method for Simultaneously Determining the Expression of Common Cancer Membrane Protein Antigens and HLA Class I Using a Multiplexed Fluorescent Immunostaining System

**Figure 5:**
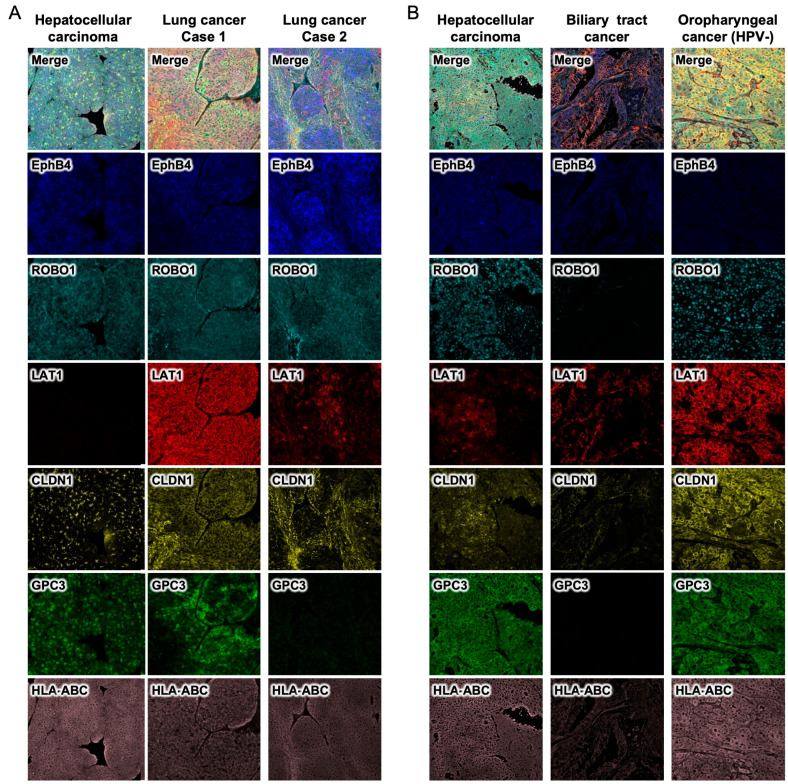
Development of a companion diagnostic method for simultaneously determining the expression of common cancer-specific antigens and HLA class I using a multiplexed fluorescent immunostaining system. Using a multiplexed fluorescent immunostaining system, we attempted to establish a companion diagnostic method for simultaneously determining the expression of 5 common cancer-specific antigens and HLA class I. We established a system for simultaneously staining five membrane protein common cancer antigens EphB4, ROBO1, LAT1, CLDN1, GPC3, and HLA class I in six colors (**A**). We evaluated the expression of these six antigens on the cell membrane using formalin-fixed paraffin sections of surgically resected specimens from actual cases of hepatocellular carcinoma, cholangiocarcinoma, and oropharyngeal carcinoma (**B**).

Using a multiplexed fluorescent immunostaining system, we attempted to establish a companion diagnostic method for simultaneously determining the expression of five common cancer membrane protein antigens and HLA class I (Figure 5). In addition, using hepatocellular carcinoma PDXs and lung cancer cases 1 and 2 PDXs, we established a system for simultaneously staining five membrane protein common cancer antigens EphB4, ROBO1, LAT1, CLDN1, GPC3, and HLA class I in six colors (Figure 5A). Although GPC3 in hepatocellular carcinoma stains cytoplasm, we were able to establish a system for simultaneously staining six membrane proteins. Next, we evaluated the expression of these six antigens on the cell membrane using formalin-fixed paraffin sections of surgically resected specimens from actual cases of hepatocellular carcinoma, cholangiocarcinoma, and oropharyngeal carcinoma (Figure 5B).

### 2.5. A Scheme for Cocktail CAR-T Cell Therapy to Overcome the Diversity of Solid Tumors

**Figure 6:**
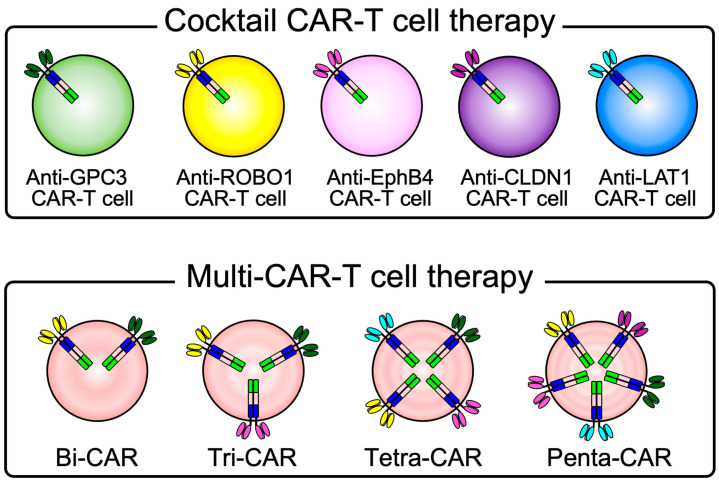
A scheme for cocktail CAR-T cell therapy to overcome the diversity of solid tumors. If cocktail CAR-T cell therapy is developed that targets multiple of the five common membrane-expressed cancer antigens, it will likely be usable against most solid tumors.

If cocktail CAR-T cell therapy is developed that targets multiple of the five common membrane-expressed cancer antigens, it will likely be usable against most solid tumors (Figure 6).

## 3. Discussion

Genetically modified T cell therapy includes CAR-T cell therapy and TCR-T cell therapy. CAR-T can only target cell membrane protein antigens. Although CD19 CAR-T has been highly effective against B-cell blood cancers [6,7,8], there are few reports of successful treatments for solid cancers [9,10,11,12,13,14], probably because there are few protein antigens specifically expressed on the surface of the cancer cell membrane.

The key to developing a groundbreaking CAR-T cell therapy effective against solid cancers is to “overcome the heterogeneity of cancer antigens”. For this purpose, it is necessary to target multiple cancer antigens simultaneously. In addition, to develop a CAR-T therapy that can be applied to many people, it is thought that the cancer antigens to be targeted are common cancer antigens expressed on the cell membrane in frequent cancers. We have selected five common membrane protein cancer antigens, including GPC3, which have high cancer specificity and are frequently expressed in various cancers.

CAR-T cell therapy targeting GPC3 has also been developed worldwide, but dramatic efficacy has not yet been reported [10,14]. GPC3 is frequently expressed in hepatocellular carcinoma and hepatoblastoma, but it is often expressed in the cytoplasm, and this study also revealed that it is expressed on the cell membrane less frequently. This is thought to be the reason why GPC3 CAR-T cell therapy is struggling. The response rate is thought to increase if treatment is limited to cancer patients who express GPC3 on the cell membrane. ROBO1, like GPC3, has been identified as a membrane protein frequently expressed in hepatocellular carcinoma [16]. Recently, CAR-T cell therapy for glioblastoma has been developed [15]. Future clinical development is expected. EphB4 has been reported to be a membrane protein expressed in breast cancer, head and neck cancer, and colorectal cancer, although its expression in normal tissues is limited [19,20,21]. Yagyu et al. developed CAR-T cell therapy targeting EphB4 [18,23]. Clinical trial results are expected. CLDN1 is a tight junction protein reported in colorectal, oral, ovarian, and lung cancers [24,25,26,27], but no cancer treatments, including CAR-T cell therapy, targeting it have yet been developed. LAT1 is the first identified light chain (lc) of the CD98 cluster, associated with the heavy chain (hc) of CD98, and is expressed on the surface of various tumor cells regardless of their origin. LAT1 is a 12-transmembrane protein, and its immunogenic extracellular domain is very small, making it difficult to make a specific monoclonal antibody (mAb) [28,29,30,31]. No CAR-T cell therapy targeting it has yet been developed.

Most CAR-T therapies currently under development target only one antigen. It has become clear that even in patients who have achieved remission with CD19 CAR-T, recurrence of CD19-negative cancer may occur [32], and it has been said that it is necessary to target multiple antigens to increase efficacy. One of the five types of membrane protein common cancer antigens is expressed in most cancer tissues. This means that if a cocktail CAR-T therapy that combines multiple CARs against these antigens can be developed, it can be used as a treatment for most solid cancers. To create a new original CAR-T that targets these five types, we are conducting joint research with Optieum Biotechnologies Inc.(Toon, Japan), which has a technology: “Eumbody System™” to create a CAR-T cell library based on a single-chain antibody (scFv) library and screen CAR-T cells with optimal therapeutic effects from the library source. We have already identified some promising CARs that target these membrane protein common cancer antigens and plan to obtain promising CARs that target the remaining membrane protein common cancer antigens one after another in the future. In addition, the patient’s own T cells are usually collected by apheresis. CAR is introduced by a viral vector, which is time-consuming and costly to manufacture, making it inefficient. Recently, the risk of secondary T cell blood cancer in CAR introduction systems using retroviruses and lentiviruses has become apparent [33], shaking up the industry. In other words, the current manufacturing method has problems with time, cost, and safety. This is also true for allogeneic cells using iPS cells [34], and it cannot be said that these problems have been eliminated even with CAR introduction systems using non-viral vectors [35].

We aim to develop a CAR-T cell therapy that prioritizes simplicity and safety while being effective. We aim to research and develop a system that does not use viral vectors but instead uses gene introduction of CAR in T cells with transient mRNA expression, allowing for repeated administration of smaller cells than usual. Safety is ensured by transient expression, and efficacy is ensured by repeated administration. In other words, by not using viral or non-viral vectors as a system for gene transfer of each CAR into T cells, the risk of secondary T-cell blood cancer—which is currently a problem—is eliminated. Furthermore, even if the target is a cancer-specific common cancer antigen, expression in normal tissues and cells is not completely absent, and by making the expression transient, safety is maximized. Furthermore, by preparing a repertoire of mRNA for many types of CARs off-the-shelf in advance, time and costs can be significantly reduced. Although there may be concerns about whether efficacy can be guaranteed because of the emphasis on safety and simplicity, this can be compensated for by being able to target many types of targets and by repeated administration.

To overcome the heterogeneity of individual cancers and apply the antibody to a wide range of solid cancer patients, we collected five common cancer membrane protein antigens, such as GPC3. In this study, we performed immunohistochemical analysis of various solid cancer specimens using antibodies against ROBO1 [15,16,17], EphB4 [18,19,20,21,22,23], CLDN1 [24,25,26,27], and LAT1 [28,29,30,31] in addition to GPC3, which we have previously studied. These antigens were frequently expressed in various solid cancers but shown to be rarely expressed, with some exceptions, in non-cancerous normal organs adjacent to the cancer. In addition, it was revealed that the cell membrane antigens EphB4, CLDN1, and LAT1 are frequently expressed in various solid cancers. CAR-T cell therapy targeting these antigens may be a savior for many solid cancer patients. In this study, we clarified the expression profiles of five common cancer membrane protein antigens collected to develop a cocktail of CAR-T cell therapies that can be applied to most solid tumor patients. Our CAR-T therapy targeting five common membrane protein cancer antigens—EphB4, CLDN1, LAT1, ROBO1, and GPC3—may be effective against many solid tumors, including childhood cancers. We will continue to develop cocktail CAR-T cell therapies that target these multiple common cancer membrane protein antigens.

## 4. Materials and Methods

### 4.1. Clinical Samples

The subjects of this study were Stage 2–4 head and neck cancer, breast cancer, esophageal cancer, lung cancer, gastric cancer, liver cancer, biliary tract cancer, pancreatic cancer, colorectal cancer, renal cell carcinoma, ovarian cancer, uterine cancer, various pediatric cancers (neuroblastoma, germ cell tumor, nephroblastoma: Wilms tumor, hepatoblastoma), and melanoma cases surgically resected at the National Cancer Center Hospital East, Kyoto Prefectural University of Medicine Hospital, Chiba University Hospital, and Kumamoto University Hospital from 1 January 2000 to 31 August 2024 as well as stage 1 gastric cancer cases endoscopically resected at the National Cancer Center Hospital East. Among these, cases in which thin-section specimens could be prepared from formalin-fixed paraffin-embedded blocks were the subjects of this study. The study was conducted with the approval of the research ethics committees of each institution (approval number 2020-352 from the National Cancer Center Research Ethics Committee). To facilitate comparison and analysis, we used specimens that contained cancerous and non-cancerous areas in the same block. Written informed consent was obtained from the patients, and the Declaration of Helsinki was used to conduct the study.

### 4.2. Immunohistochemical Analysis of Expression of Five Kinds of Common Cancer Membrane Protein Antigens

Immunohistochemical analysis was performed on 4 μm paraffin sections prepared from formalin-fixed paraffin blocks of human cancer tissues. Commercially available microarray slides of normal tissues were used (BioChain Institute Inc., Newark, CA, USA). Deparaffinization was performed using xylene and ethanol, and endogenous peroxidase was inactivated using 0.3% H_2_O_2_/methanol. Antigen retrieval was performed using Target Retrieval Solution, pH 9 (Agilent Technologies, Santa Clara, CA, USA) or heat treatment in a pressure cooker or microwave in AR6 (Akoya Biosciences, Marlborough, MA, USA) inactivation buffer. After blocking with porcine serum, primary antibodies against each antigen were reacted. Glypican-3 (GPC3) (BioMoosaics, Burlington, VT, USA, 300-fold dilution), roundabout guidance receptor 1 (ROBO1) (Proteintech, Rosemont, IL, USA, 300-fold dilution), Ephrin type-B receptor 4 (EphB4) (Cell Signaling Technology, Inc., Danvers, MA, USA, 300-fold dilution), Claudin 1 (CLDN1) (Cell Signaling Technology, Inc., Danvers, MA, USA, 300-fold dilution), L-type amino acid transporter 1 (LAT1) (abcam, Cambridge, UK, 200-fold dilution). The secondary antibody was used as polymer reagent (EnVision+ System-HRP Labelled Polymer Anti-Rabbit or EnVision+ System-HRP Labelled Polymer Anti-Mouse (Agilent, Santa Clara, CA, USA). The Liquid DAB+ Substrate Chromogen System (Agilent, Santa Clara, CA, USA) was used for color development. After counterstaining with hematoxylin, the specimens were dehydrated and cleared using ethanol and xylene, and the specimens were made into virtual slides using a NanoZoomer 2.0HT (NDP.scan ver.2.5-ver.3.2) and S360 (NZAcquire ver.3.1) (Hamamatsu Photonics, Hamamatsu, Japan). The intracellular localization of tumor tissue was also evaluated by immunohistochemical analysis. The presence and localization (whether cell membrane or not) of staining were evaluated in tumor cells for each case. Three researchers analyzed immunohistochemical staining with these methods.

### 4.3. Multiplex Fluorescence Immunohistochemical Staining and Analysis of Cancer Antigens and HLA Class I

Multiplex fluorescent immunohistochemistry (MFIH) was performed on 4 μm thick tissue sections of various cancers using the PerkinElmer Opal kit. Fluorescent multiplex histochemistry images were acquired using a tissue section quantitative analysis imaging system (Vectra 3, Akoya Biosciences, Marlborough, MA, USA), and up to 20 regions of interest (ROIs) (699 × 500 μm) were randomly selected in the center and margin of each tumor and evaluated using an image analysis program (Inform 2.6., PerkinElmer, Inc., Shelton, CT, USA). The distribution of specific cancer antigens was also analyzed. HLA class I and five common cancer membrane protein antigens (CLDN1, EphB4, LAT1, ROBO1, and GPC3) were selected as objects for MFIH.

## Figures and Tables

**Table 1:** Expression of five kinds of common cancer antigens on the cell membrane in various solid cancers.

Common Cancer Antigens *	CLDN1	EphB4	LAT1	ROBO1	GPC3
Head and neck cancer	44/44 (100)	53/56 (95)	41/44 (93)	13/44 (30)	3/57 (5)
oral cancer	14/14 (100)	20/20 (100)	14/14 (100)	2/14 (14)	0/21 (0)
pharyngeal cancer	30/30 (100)	33/36 (92)	27/30 (90)	11/30 (37)	3/36 (8)
Breast cancer	7/36 (20)	30/36 (83)	15/36 (42)	2/35 (6)	0/36 (0)
Esophageal cancer	15/16 (94)	15/16 (94)	15/16 (94)	6/16 (38)	1/16 (6)
Lung cancer	45/72 (63)	57/72 (79)	49/72 (68)	19/72 (26)	4/72 (6)
adenocarcinoma	20/45 (44)	35/45 (78)	22/45 (49)	5/45 (11)	1/45 (2)
squamous cell carcinoma	25/27 (93)	22/27 (81)	27/27 (100)	14/27 (52)	3/27 (11)
Gastric cancer	14/29 (48)	27/29 (93)	12/29 (41)	0/29 (0)	0/19 (0)
advanced	8/10 (80)	10/10 (100)	8/10 (80)	0/10 (0)	NT **
early	6/19 (32)	17/19 (89)	4/19 (21)	0/19 (0)	0/19 (0)
Biliary cancer	26/28 (93)	24/30 (80)	10/20 (50)	6/29 (21)	0/20 (0)
intrahepatic	7/8 (88)	10/10 (100)	NT	2/10 (20)	NT
extrahepatic	10/10 (100)	7/10 (70)	4/10 (40)	3/10 (30)	0/10 (0)
gallbladder	9/10 (90)	7/10 (70)	6/10 (60)	1/9 (11)	0/10 (0)
Pancreatic cancer	11/11 (100)	9/12 (75)	4/13 (31)	0/11 (0)	0/12 (0)
Hepatocellular carcinoma	13/20 (65)	14/20 (70)	0/14 (0)	14/19 (74)	1/20 (5)
Colorectal cancer	15/15 (100)	14/15 (93)	15/15 (100)	0/15 (0)	0/15 (0)
Renal cell carcinoma	3/19 (16)	0/19 (0)	3/19 (16)	2/19 (11)	0/19 (0)
Ovarian cancer	17/18 (94)	15/18 (83)	12/18 (67)	0/18 (0)	1/18 (6)
serous adenocarcinoma	16/17 (94)	14/17 (82)	11/17 (65)	0/17 (0)	0/17 (0)
clear cell carcinoma	1/1 (100)	1/1 (100)	1/1 (100)	0/1 (0)	1/1 (100)
Uterine cancer	7/7 (100)	6/7 (86)	7/7 (100)	1/7 (14)	0/7 (0)
Pediatric cancer	15/33 (45)	13/33 (39)	14/33 (42)	16/32 (50)	9/33 (27)
hepatoblastoma	9/9 (100)	8/9 (89)	2/9 (22)	6/9 (67)	3/9 (33)
nephroblastoma	4/10 (40)	3/10 (30)	3/10 (30)	8/10 (80)	5/10 (50)
germ cell tumors	2/6 (33)	2/6 (33)	6/6 (100)	1/6 (17)	1/6 (17)
neuroblastoma	0/8 (0)	0/8 (0)	3/8 (38)	1/7 (14)	0/8 (0)
Melanoma	5/20 (25)	13/20 (65)	11/20 (55)	2/20 (10)	0/20 (0)
ALM	0/7 (0)	4/7 (57)	3/7 (43)	0/7 (0)	0/7 (0)
MM	0/5 (0)	5/5 (100)	4/5 (80)	2/5 (40)	0/5 (0)
SSM	3/5 (60)	3/5 (60)	3/5 (60)	0/5 (0)	0/5 (0)
LMM	2/3 (67)	1/3 (33)	1/3 (33)	0/3 (0)	0/3 (0)
Total	237/368 (64)	290/383 (76)	208/356 (58)	81/366 (22)	19/364 (5)

* CLDN1: Claudin 1, EphB4: Ephrin type-B receptor 4, LAT1: L-type amino acid transporter 1, ROBO1: Roundabout Homolog-1, GPC3: glypican-3; ** NT: not tested.

## Data Availability

The data presented in this study are available on request from the corresponding author.

## Abbreviations

The following abbreviations are used in this manuscript:

CAR	chimeric antigen receptor
GPC3	glypican-3
ROBO1	roundabout guidance receptor 1
EphB4	Eph receptor B4
CLDN1	claudin-1
LAT1	L-type amino acid transporter 1
